# Direct Synthesis of Vertical Self-Assembly Oriented Hexagonal Boron Nitride on Gallium Nitride and Ultrahigh Photoresponse Ultraviolet Photodetectors

**DOI:** 10.3390/nano13091546

**Published:** 2023-05-05

**Authors:** Yi Peng, Yufei Yang, Kai Xiao, Yanlian Yang, Haoran Ding, Jianyu Deng, Wenhong Sun

**Affiliations:** 1 Research Center for Optoelectronic Materials and Devices, Guangxi Key Laboratory for the Relativistic Astrophysics, School of Physical Science & Technology, Guangxi University, Nanning 530004, China; 2 State Key Laboratory of Featured Metal Materials and Life-Cycle Safety for Composite Structures, Nanning 530004, China

**Keywords:** GaN-based, vertically ordered h-BN, UV photodetectors

## Abstract

The applications of three-dimensional materials combined with two-dimensional materials are attractive for constructing high-performance electronic and photoelectronic devices because of their remarkable electronic and optical properties. However, traditional preparation methods usually involve mechanical transfer, which has a complicated process and cannot avoid contamination. In this work, chemical vapor deposition was proposed to vertically synthesize self-assembly oriented hexagonal boron nitride on gallium nitride directly. The material composition, crystalline quality and orientation were investigated using multiple characterization methods. Thermal conductivity was found to be enhanced twofold in the h-BN incorporated sample by using the optothermal Raman technique. A vertical-ordered (VO)h-BN/GaN heterojunction photodetector was produced based on the synthesis. The photodetector exhibited a high ultraviolet photoresponsivity of up to 1970.7 mA/W, and detectivity up to 2.6 × 10^13^ Jones, and was stable in harsh high temperature conditions. Our work provides a new synthesis method to prepare h-BN on GaN-based materials directly, and a novel vertically oriented structure of VO-h-BN/GaN heterojunction, which has great application potential in optoelectronic devices.

## 1. Introduction

Hexagonal boron nitride (h-BN) has a graphene-like structure with high mechanical strength, thermal conductivity, and chemical stability. The synthesis methods, properties, and potential applications of h-BN have been comprehensively reported in the literature [1,2,3,4]. The development of combining h-BN with materials such as Si and GaN appeared on the scene in applications of field emission devices [5], graphene/h-BN/n-Si heterojunction photovoltaic cells [6], and h-BN/GaN photoelectronic devices such as light-emitting diodes (LEDs) and UV photodetectors [7,8]. Another h-BN application is in thermal management to improve the performance of GaN-based high electron mobility transistors, which takes advantage of its excellent in-plane thermal conductivity and electrical insulation [9,10,11].

Conventional synthesis strategies of h-BN on GaN applications focus on the transfer of h-BN from that grown on Cu foils by metal–organic vapor phase epitaxy (MOVPE) [11,12,13]. This suffers the disadvantages of contamination and size limitation during sample transfer and stacking. Therefore, it is imperative to develop another way to synthesize h-BN. Some researchers tried to prepare h-BN thin films on AlGaN/GaN HEMTs directly using microwave plasma enhanced chemical vapor deposition (MWPECVD), and found that the c-axis of h-BN is preferentially oriented perpendicular to the surface of the sample [9]. To take advantage of the superior in-plane properties of h-BN, such as high heat dissipation and electron mobility, vertically ordered (VO) h-BN prepared with high-power impulse magnetron sputtering deposited on GaN-based HEMTs was studied by one research group [14]. However, these equipment systems are complex and expensive. Additionally, some other vertically ordered 2D materials such as MoS_2_ also showed attraction in the application of optoelectronic devices [15]. Therefore, more convenient and economical methods of synthesis of VO h-BN and their applications are worth investigating.

To evaluate h-BN’s thermal performance, it is critical to find a convenient way to measure the change in thermal conductivity of GaN-based materials before and after h-BN is incorporated. There are two major categories of methods to measure thermal conductivity: steady state and transient [16]. Most of those methods have a specified dimension with a well-defined thickness, which is not suitable for thin film materials. Among the steady-state techniques, optothermal Raman (OTR) was introduced to measure the thermal conductivity of thin film materials, from ultra-thin graphene to the constituent layers in tristructural-isotropic (TRISO) particles, as a fast, nondestructive testing technique [17,18,19]. OTR is a swift and convenient technique combining the principle that the Raman mode is strongly temperature-dependent and the fact that the Raman laser can act as a heat source for local temperature rise [20]. However, the feasibility of OTR’s application in nitride semiconductor materials needs further study.

In this article, we report the preparation of large-area vertically ordered (VO) h-BN using chemical vapor deposition (CVD). The deposition was achieved by employing Boron powder and ammonia gas as the sources of boron and nitrogen, respectively. The Van der Waals (VDW) force is generally weaker than typical chemical bonds, with an interaction energy (10^−1^ to 10^1^ kJ/mol) two to three orders of magnitude lower than that of the ionic or covalent bonds (10^2^–10^3^ kJ/mol) [21]. The breakage of covalent bonds between Ga and N under appropriate temperatures provides stronger chemical activity on the interface to attract B atoms that lead to the synthesis of vertically self-ordered oriented h-BN on GaN directly. Using the optothermal Raman technique, the thermal characteristics of VO-hBN are evaluated conveniently and rapidly. Furthermore, the optoelectronic devices based on the VO-hBN/GaN heterojunction that were prepared by the new method are studied.

## 2. Materials and Methods

**Figure 1:**
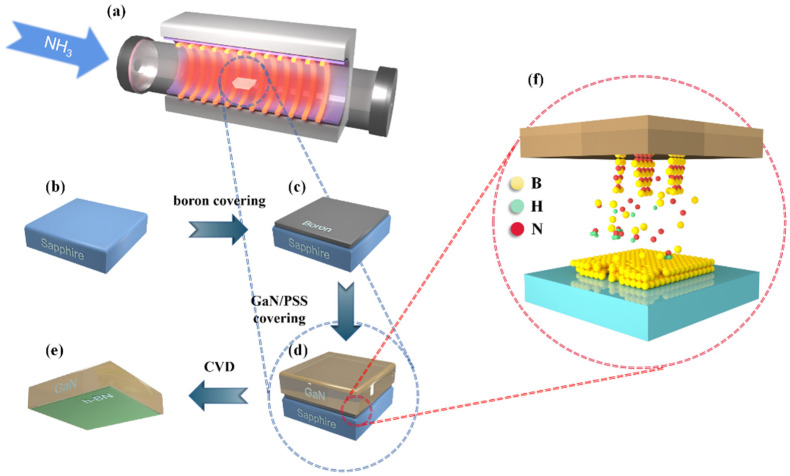
Schematic diagrams of the CVD system used for vertical orientation h-BN growth and the process of the synthesis. (**a**) The reaction equipment of CVD; (**b**) the preparation of sapphire substrate before process; (**c**) the process of covering the sapphire substrate with boron; (**d**) the process of covering the prepared B/sapphire sample with the GaN epilayer; and (**e**) the final synthetic sample after B/sapphire substrate removed; (**f**) the microprocess of the synthesis.

GaN epilayers with a thickness of 2 μm were grown on (0001) patterned sapphire substrates (PSS) using low-pressure metalorganic chemical vapor deposition (LP-MOCVD) with trimethylgallium and NH_3_ as sources, and hydrogen and nitrogen as carrier gases. The GaN epilayer was n-type doped by Si with a carrier concentration of 5 × 10^17^ cm^−3^. The h-BN was prepared on the GaN epilayer directly with Boron and NH_3_ (99.9995%) as the source at 1100 °C in the furnace demonstrated in Figure 1a. The boron powder (48μm) was used as an evaporator source for using the E-beam to deposit boron on sapphire, firstly (shown in Figure 1b,c). Using the GaN epilayer to cover the prepared B/sapphire sample (as Figure 1d shows; the PSS layer of GaN is not shown), Figure 1d,f show the final synthetic sample and the microprocess of synthesis, respectively. The flow of NH_3_ was 100 sccm in the whole process, the rate of temperature climbing up was 10 °C/min, and the chemical reaction lasted 3 h with a constant 1100 °C temperature. The exhaust was absorbed by H_2_O.

The Raman measurements were carried out using a HORIBA labRAM HR Evolution spectrometer with optical excitation from a 325 nm 30 mW He-Cd laser and 532 nm 100 mW DPSS laser. The grating was chosen as 1800 grooves/mm. The signals were collected with a charge-coupled device (CCD) array. All the Raman spectra were recorded in back-scattering geometry from the sample surface with the light propagating parallel to the sample surface. The scattered light was not analyzed for its polarization. The samples were sealed in a LinKam cold/hot stage (with continuous liquid nitrogen flow), which was placed under the microscope of this setup and provided an environment of samples that varied in temperature range between 77 and 330 K. The sample temperature was measured with a K-type thermocouple with an accuracy of less than ±1 K. The laser duty cycle was controlled at 0.01%, 0.1%, 1%, 3.2%, 5%, 10%, 25%, 50%, and 100%. The laser beam was focused onto a spot with a diameter of about 1 μm at the sample surface by using a microscope system. For the analysis of the spectra, Gauss fitting was used to determine the position and integrated intensity of the peaks. The combination of the temperature-dependence and power-dependence Raman could reveal the change in thermal conductivity within the process. On the other hand, the result of Raman mapping helped to comprehend the uniformity of h-BN synthesized.

The XPS measurements were taken using Thermo Scientific K-Alpha with A1 K_α_ (hυ = 1486.6 eV) as the excitation source, and the working voltage and current were 12 kV and 6 mA, respectively. The vacuum level was kept as less than 5.0 × 10^−7^ mBar, and the binding energy of all elements was calibrated according to C1s = 284.80 eV. The result of the XPS was helpful to study the formation of h-BN.

PANalytical X’pert3 MRD equipped with Ge [220] four-crystal monochromator in a triple-axis model (Cu K_α1_ = 1.5406 Å) was employed to acquire the results of high-resolution X-ray diffractometer (HRXRD) for the investigation of the quality change of the GaN epilayer before and after h-BN growth.

High resolution transmission electron microscopy (HRTEM, FEI Talos F200X) investigations were carried out with a 200 keV to research the epitaxial relationships between GaN and h-BN and the ordered orientation of h-BN with the support shown in selected area electron diffraction (SAED).

The current–voltage (I–V) characteristics of the device and the time-dependence of the photocurrent (I-t) were measured with a semiconductor parameter analyzer (Keithley 2400), and commercial LED light at a wavelength of 255 nm, 280 nm, and 310 nm was the UV source. The incident power density generated by the LED light on the sample surface was measured to be 1.5 mW/cm^2^.

Other equipment involved in this experiment included TU-1901 VU-Vis (Beijing General Analytical Instrument) for transmissivity measurement, TENSOR II (BRUKER) for FTIR measurement, and LFA 427 Laser Flash Apparatus (NETZSCH) for thermal diffusivity and conductivity measurement. It is worth noting that the direction of measurement for thermal diffusivity is perpendicular to the surface of the samples.

## 3. Results and Discussion

**Figure 2:**
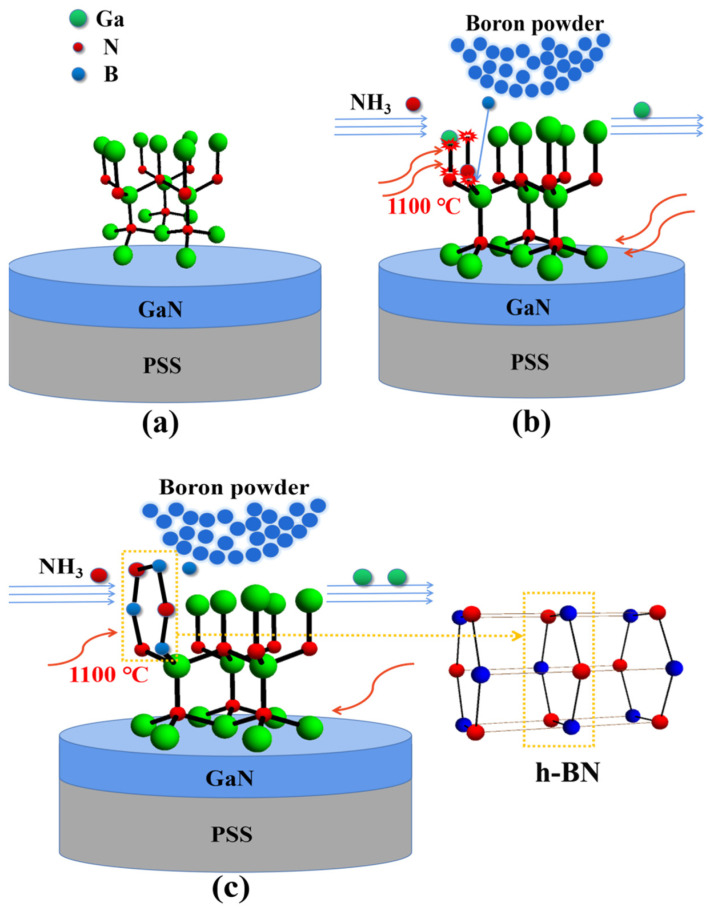
Raman spectra of the RTA-treated B-ion implanted GaN in the temperature range of 80–300 K. (**a**) the (002) crystal orientation GaN of the classical wurtzite structure epitaxially grown on PSS; (**b**) the dangling bonds appeared with Ga-N bonds broken at 1100 °C; (**c**) the self-ordered oriented h-BN formed in GaN.

Figure 2 describes a theoretical hypothesis of the intrinsic reaction at the atomic level during the experiment shown in Figure 1. Figure 2a shows the (002) crystal orientation GaN of the classical wurtzite structure epitaxially grown on PSS. Figure 2b suggests that the dangling bonds appeared with Ga-N bonds broken, considering that the temperature of this experiment went up to 1100 °C. Due to the lack of out-of-plane dangling bonds in h-BN, and considering the previous studies where the small size of the B atom makes it possible to incorporate B on the N site in GaN [22], a physical model used to illustrate how the self-ordered oriented h-BN formed in GaN is shown in Figure 2c.

**Figure 3:**
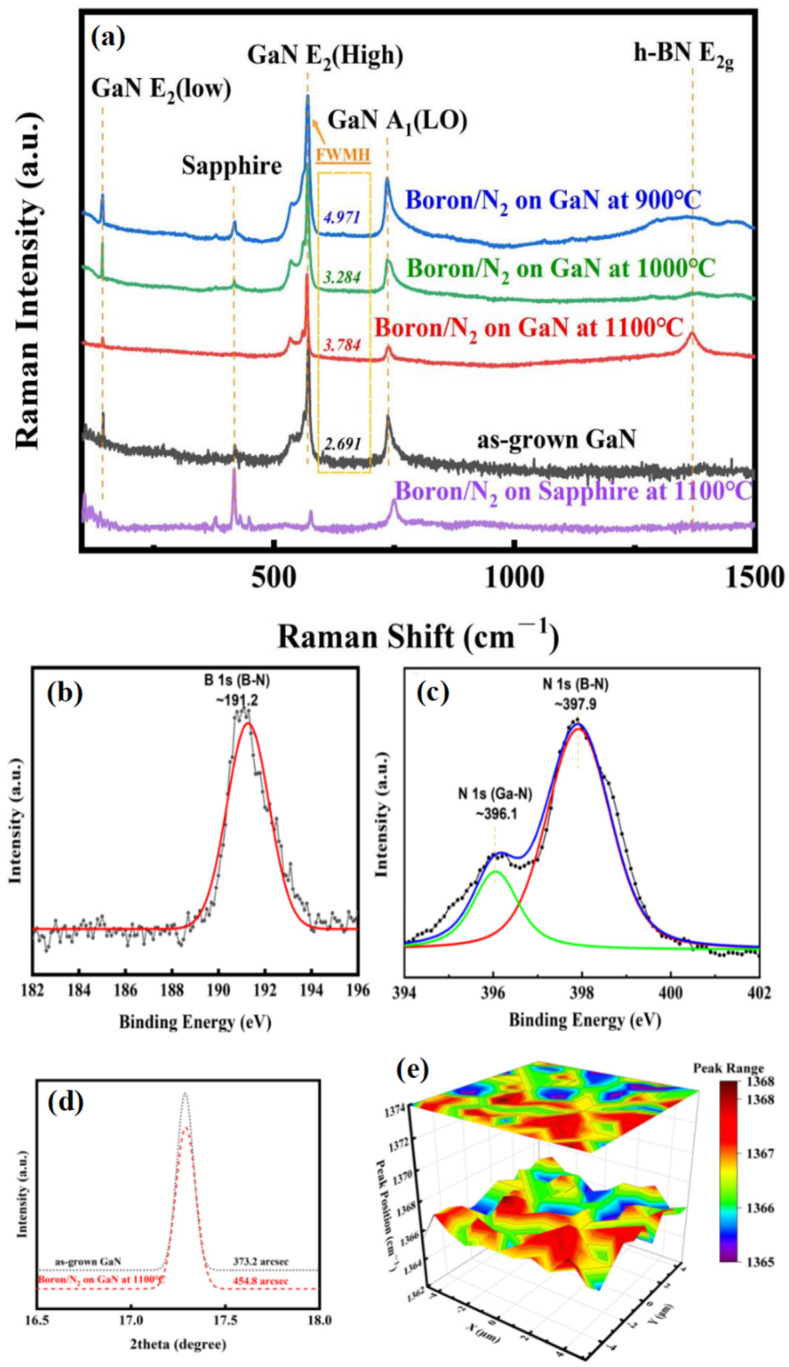
(**a**) Comparison of the Raman spectra of different substrates and growth temperature for h-BN samples synthesized; high-resolution XPS spectra of (**b**) B 1s and (**c**) N 1s of VO h-BN/GaN (B/GaN1100 sample); (**d**) comparison of the XRD rocking curve of as-grown GaN (As-GaN sample) and VO h-BN/GaN (B/GaN1100 sample); and (**e**) Raman mapping in an area of 10 × 10 μm^2^ of VO h-BN/GaN (B/GaN1100 sample).

To confirm the hypothesis in Figure 2, multiple characterizations were employed in Figure 3. The Raman spectra of the sample processing with different reaction conditions or substrates is illustrated in Figure 3a. We used As-GaN, B/GaN900, B/GaN1000, B/GaN1100, and B/Sap1100 to abbreviate the corresponding samples of as-grown GaN, Boron/N_2_ on GaN at 900 °C, Boron/N_2_ on GaN at 1000 °C, Boron/N_2_ on GaN at 1100 °C, and Boron/N_2_ on sapphire at 1100 °C in the full text below. The E_2_ and A_1_(LO) modes of GaN are Raman active with the configuration of $Z(X,-)\overset{-}{Z}$ [23], and were clearly distinguishable in all GaN-based samples. The E_2_g Raman mode (~1367 cm^−1^) of h-BN only appeared in sample B/GaN1100, whose process temperature was close to the temperature of GaN thermal decomposition [24]. The different FWHM of the GaN E_2_(high) mode are presented beside the peak, from which can be inferred the evolution of the quality of the GaN layer. The detailed analysis will be discussed with the XRD results in Figure 3d later.

In order to further prove the formation of h-BN, the results of XPS, shown in Figure 3b,c were employed to analyze the surface composition of the B/Sap1100 sample. The binding energies for the B 1s and N 1s peaks, determined from the XPS spectra, were 191.2 (Figure 3a) and 397.9 eV (Figure 3b), respectively. These values matched with those previously reported for h-BN [25]. Another N 1s peak at 396.1 eV in Figure 3b originated from GaN [26].

The quality of the h-BN/GaN was estimated on the basis of two parts: the XRD rocking curve of GaN before and after the deposition of h-BN, and the Raman mapping of h-BN. Figure 3d shows the XRD rocking curve of As-GaN and B/GaN1100; their full widths at half maximum (FWHM) were 373.2 arcsec and 454.8 arcsec, respectively. The FWHM was found to broaden by 21.9% with the h-BN deposition, consistent with the change in Raman in Figure 3a, which indicates that the quality of the GaN substrate decreases slightly due to the breakage of covalent bonds between Ga and N under 1100 °C. Figure 3e displays the Raman mapping of the h-BN layer in an area of 10 × 10 μm^2^. The E_2g_ Raman mode of h-BN detected for all of the point measures in that area was in the range from 1365 to 1368 cm^−1^, which confirms the uniformity of the h-BN distribution on GaN.

**Figure 4:**
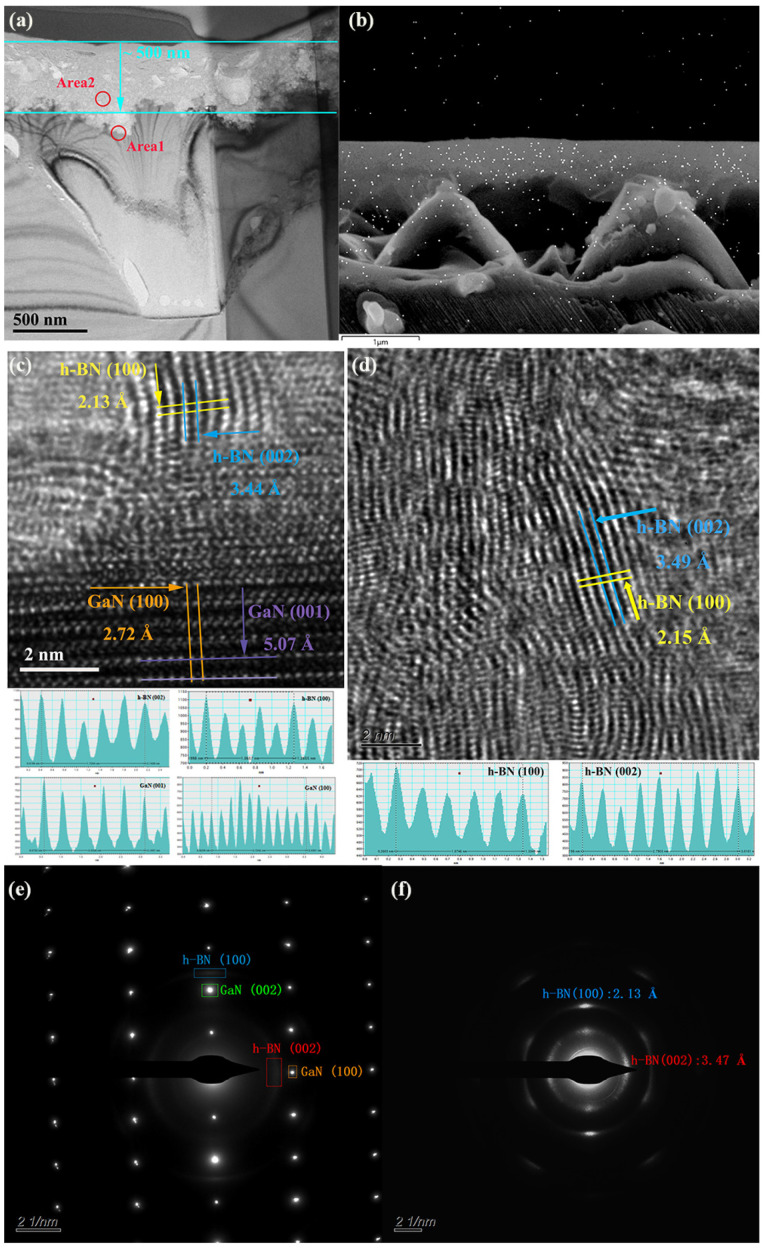
(**a**) The low magnification cross-section B/GaN1100 sample TEM image of a VO h-BN/GaN synthesis layer with a thickness around 500 nm; (**b**) the EDS spectrum of cross-section B/GaN1100 sample for Boron. The HRTEM image of (**c**) the juncture of h-BN and GaN (relates to “area1” in (**a**)) and (**d**) inside the synthesis layer of h-BN (relates to “area2” in (**a**)); and the SAED images of (**e**) the juncture of h-BN and GaN (relates to “area1” in (**a**)) and (**f**) inside the synthesis layer of h-BN (relates to “area2” in (**a**)).

Figure 4a shows the TEM image with a low magnification; a synthesis layer with a thickness of ~500 nm was observed distinctly. The “area1” includes the mixed part of the synthesis layer and substrate layer, and “area2” is completely inside the synthesis layer. The EDS spectrum of the cross-section of the B/GaN1100 sample for Boron is exhibited in Figure 4b, in which Boron can be distinguished as a concentrated distribution in the synthesis layer of ~500 nm.

The orientation of h-BN was studied in Figure 4c–f. The HRTEM and SAED images, including the mixed part of h-BN and GaN (related to “area1”), are shown in Figure 4c,e, respectively. Two significantly different sets of interplanar distances and crystal orientations could be obtained from the HRTEM image. The interplanar distances 2.72 Å and 5.07 Å were indexed as (1 0 0), and (0 0 1) crystal planes of wurtzite GaN that had typical values of 2.76 Å and 5.18 Å, respectively. In addition, it is clear that the (0 0 1) crystal planes of wurtzite GaN were parallel to the surface of the sample. On the wurtzite GaN layer, another set of interplanar distances with values of 2.13 Å, and 3.44 Å could be assigned to (1 0 0) and (0 0 2) crystal planes of h-BN, whose typical values were 2.17 Å and 3.33 Å, respectively. The difference compared to the pure GaN layer is that the (0 0 2) crystal planes of the h-BN layer are perpendicular to the surface of the sample. The SAED image of this area provides the same information. Figure 4d,f show the HRTEM and SAED images, respectively, inside the synthesis layer (relating to “area2”), and only the interplanar distances of h-BN were obtained. The SAED image of h-BN inside the synthesis layer has a clearer signal than those on the boundary, which suggests that the crystal lattice is more orderly in that area.

**Figure 5:**
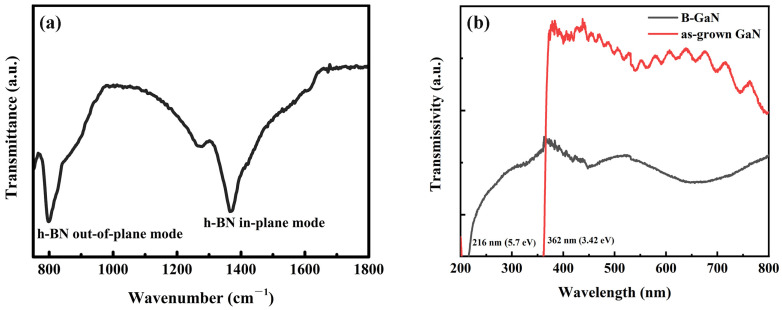
(**a**) The FTIR spectra and (**b**) the UV–Vis transmission spectra of VO h-BN/GaN (B/GaN1100 samples).

Fourier Transform Infrared Spectrometer (FTIR), which has been widely used for BN characterization, can also be applied for differentiating the crystal orientation of h-BN by providing information about the vibrational modes in it. The typical FTIR image of h-BN has a peak at ~800 cm^−1^ (out-of-plane B-N-B bending mode absorption peak) and another peak at ~1380 cm^−1^ (in-plane B-N stretch mode) [27]. Technically, h-BN planes exhibit an Infrared (IR) active mode at 800 cm^−1^, which is forbidden in the case of an IR beam with normal incidence. Therefore, when FTIR measures along the c-axis of h-BN, the in-plane mode is usually dominant. However, when the measurement is perpendicular to the c-axis, the out-of-plane mode is expected to intensify gradually [28]. In this work, the signals of the in-plane mode and the out-of-plane mode were recorded at 798.5 cm^−1^ and 1369.4 cm^−1^ (shown in Figure 5a), respectively. The ratio between the integrated intensities of the two modes was 69.73% (I_out_/I_in_). This provides evidence that the h-BN is vertically synthesized on GaN on a larger scale. The absorption edges of 5.9 eV in transmissivity measurements performed by UV–vis also suggest the formation of h-BN (shown in Figure 5b).

**Figure 6:**
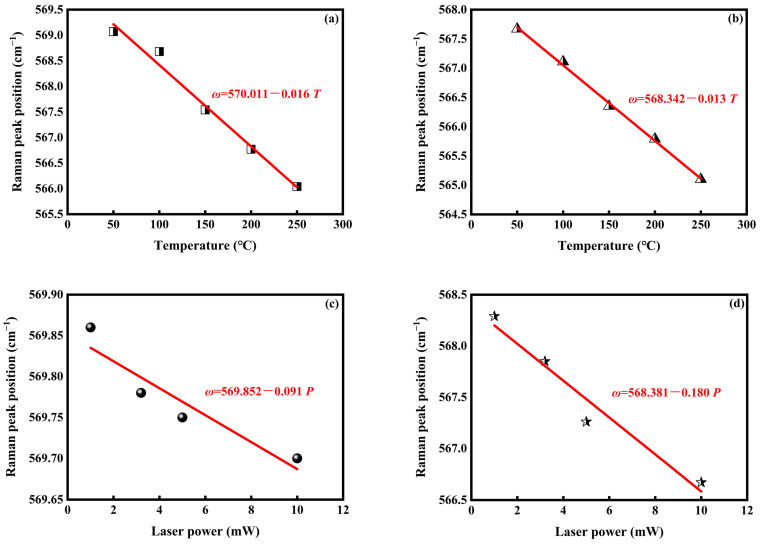
Temperature (*T*) dependence of the Raman peak position (*ω*) measured for (**a**) B/GaN1100 (*dω/dT*) and (**b**) As-GaN (*dω/dT*), and power (*P*) dependence of the Raman peak position (*ω*) measured for (**c**) B/GaN1100 (*dω/dP*) and (**d**) As-GaN (*dω/dP*).

The excellent in-plane thermal management properties of h-BN can help to improve the performance of electron and photoelectron devices. The effect of enhancing thermal characteristics by adding vertically ordered h-BN in GaN was investigated. Thermal conductivity is a particularly important property for evaluating the thermal characteristics of the materials. To use the optothermal Raman (OTR) technique, the temperature-dependence Raman and power-dependence Raman of As-GaN and B/GaN1100 were acquired in Figure 6. The localized temperature distribution in cylindrical coordinates within the material have been previously deduced and formulated as [29]:

(1) $$T(r,z)=\frac{\alpha AP}{2\pi K}\int_{0}^{\infty }J_{0}(\lambda \frac{r}{r_{0}})exp(-\frac{\lambda^{2}}{4})\times \frac{\alpha r_{0}exp(-\lambda \frac{z}{r_{0}})-\lambda exp(-\alpha z)}{{\left(\alpha r_{0}\right)}^{2}-\lambda^{2}}d\lambda$$

where *λ* is a variable of the Bessel function of the first kind and zero order *J*_0_(*λr*), *K* is the thermal conductivity, *A* is the absorptivity, *α* is the absorption coefficient, *P* is the laser power, and *r*_0_ is the laser spot radius.

The average temperature within the probing volume of Raman spectra in the materials can be deduced from (1) using double integral [29]:

(2) $$\overset{-}{T}=\frac{1}{V_{c}}\int_{0}^{\delta }\int_{0}^{r}T(r,z)\times 2\pi rdrdz$$

where *V_c_* is the probing volume of Raman spectra in the materials, and its expression is as follows:

(3) $$V_{c}=\frac{\pi \delta }{3}({r_{0}}^{2}+r_{0}r^{\prime }+{r^{\prime }}^{2})$$

Additionally, *r*′ is the laser beam radius at a distance of *δ* below the sample surface.

The thermal conductivity of the sample can be deduced as:

(4) $$K=\frac{\alpha AP\overset{-}{N}(r,z)}{V_{c}\overset{-}{T}}$$

where $\overset{-}{N}(r,z)$ is the average temperature factor, which can be deduced from Equation (1).

The temperature- and excitation-power-dependence Raman measurements were repeated many times, and a good linear relationship between peak position and the temperature and the laser power was identified. Therefore, Equation (4) can be rewritten in differential form as:

(5) $$K=\frac{\alpha A\overset{-}{N}(r,z)dP}{V_{c}d\overset{-}{T}}=\frac{\alpha A\overset{-}{N}(r,z)(d\omega /d\overset{-}{T})}{V_{c}(d\omega /dP)}$$

Considering the low content of boron and the high bandgap energy at around 5.9 eV of h-BN, the absorption characteristics of the processing sample changed very little with the 325 nm laser source. The change in the thermal conductivity of the sample before and after the process can be compared by a simplified formula:

(6) $$K\propto \frac{\left(d\omega /d\overset{-}{T}\right)}{\left(d\omega /dP\right)}$$

where *dω/dT* is the temperature coefficient and *dω/dP* is the power coefficient derived by the Raman peak position measurement in Figure 6, and the Raman mode of GaN E_2_(high), which is the most sensitive to temperature, was used as a probe.

In order to avoid the laser heating effect, a 532 nm laser source was used in the measurements of temperature-dependent Raman. For obtaining the power coefficient by using a laser as the thermal source, a 325 nm laser was used in the measurements of power-dependent Raman.

The average temperature coefficients, the power coefficients obtained from the As-GaN and B/GaN1100, and the calculated thermal conductivity are summarized in Table 1. 

The close value of the temperature coefficients indicated that there was a very small difference in the quality and absorption characteristics between As-GaN and B/GaN1100 samples. On the other hand, the smaller power coefficient of B/GaN1100 suggested that the Raman mode position of the h-BN/GaN samples shifted more slightly than As-GaN, which benefited from better heat dissipation. Using the simplified formula in Equation (6) and the results in Table 1, the thermal conductivity of the B/GaN1100 sample was almost twice as strong as the As-GaN sample.

The thermal diffusivity measured using the laser flash technique for the As-GaN and B/GaN1100 samples is shown in Table 2 in support of the result acquired from OTR. The results show a slight difference to OTR due to different sizes and thicknesses of the thin films. However, the improvement of heat dissipation can be observed.

**Figure 7:**
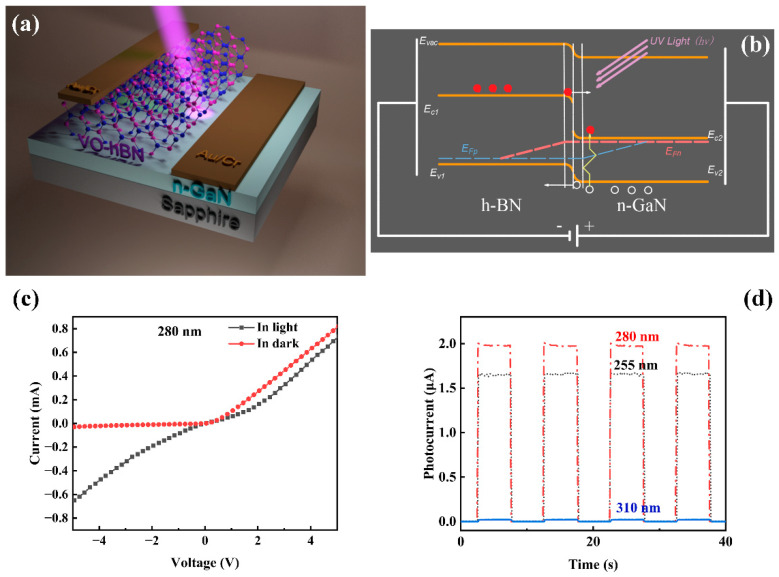
(**a**) Schematic illustration of the photoresponse of the VO-hBN/GaN heterojunction device. (**b**) Energy band diagram of VO-hBN/GaN heterojunction under UV illumination. (**c**) The typical I–V curves of VO-hBN/GaN heterojunction in dark and under light illuminations of 1.5 mW/cm^2^ for 280 nm, respectively. The insert shows the ohmic contact of the electrode. (**d**) Photocurrent as a function of time without bias voltage under illumination of 1.5 mW/cm^2^ for 255 nm, 280 nm, and 310 nm, respectively.

The UV-PDs based on VO-hBN/GaN were prepared, and the schematic structure is shown in Figure 7a. The Au/Cr conductive layer (50 nm) was deposited by magnetron sputtering as the electrodes on h-BN and GaN, respectively. Figure 7c gives the current–voltage (I–V) curves of the VO-hBN/GaN heterojunction in the dark and under commercial UV LED illumination (280 nm, 1.5 mW/cm^2^), respectively. Obviously, the device exhibited a photodiode-like behavior with a significant response to 280 nm light, which could be observed in other VO-2D/3D heterojunction structures such as V-MoS_2_/Si. Figure 7b is used to illustrate the working mechanism of VO-hBN/GaN, which can explain the photodiode-like behavior. It is worth noting that some 2D materials are naturally p- or n-doped, and h-BN was reported to be intrinsically p-type [12]. Therefore, VO-hBN and n-GaN form a p–n junction at the interface.

Upon the illumination of the photodetectors, the photo-excited carriers were driven throughout the interface of the space charge region under external reverse bias, and then the electrons were driven into the GaN side and the holes into the h-BN side, and the reverse current was increased; that was why the device exhibited a photodiode-like behavior and a significant photocurrent (shown in Figure 7c). The good ohmic contact of the electrode in the insert of Figure 7c indicates that the rectification characteristics originated from the VO-hBN/GaN heterojunction, but not the electrode. In order to explore whether this heterojunction works in a self-driven condition, photocurrent as a function of time was measured by periodically turning on and off the light (255 nm, 280 nm, 310 nm of 1.5 mW/cm^2^, respectively) without bias voltage, as shown in Figure 7d. The photocurrent excited by 255 nm or 280 nm light showed a clear change when periodically turning the light on and off, which indicates that the heterojunction meets the self-driven condition under the light source of a specific wavelength.

**Figure 8:**
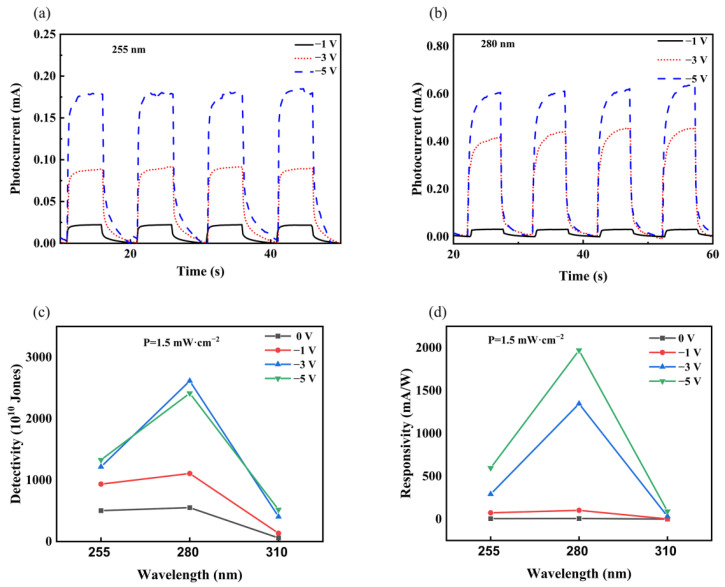
(**a**) Photocurrent as a function of time under −1 V, −3 V, and −5 V bias voltages under illumination of 1.5 mW/cm^2^ for 255 nm, respectively. (**b**) Photocurrent as a function of time under −1 V, −3 V, and −5 V bias voltages under illumination of 1.5 mW/cm^2^ for 280 nm, respectively. (**c**) Responsivity as a function of illumination wavelength under 0 V, −1 V, −3 V, and −5 V bias voltages with 1.5 mW/cm^2^ illumination, respectively. (**d**) Detectivity as a function of illumination wavelength under 0 V, −1 V, −3 V, and −5 V bias voltages with 1.5 mW/cm^2^ illumination, respectively.

For further research into the photoelectric property of VO-hBN/GaN heterojunction, photocurrent as a function of time under −1 V, −3 V, and −5 V bias voltages was shown in Figure 8a,b under the illumination of 1.5 mW/cm^2^ for 255 nm and 280 nm, respectively. When compared with the situation of zero bias voltage, the photocurrent of the device increased greatly under reverse bias voltage. Taking the most sensitive wavelength (280 nm light) for an example, the highest photoresponsivity was up to 1970.7 mA/W under a −5 V bias voltage (Figure 8c), and the highest detectivity was up to 2.6 × 10^13^ Jones under a −3 V bias voltage (Figure 8d).

The result of responsivity, shown above, can be determined with Equation (7):

(7) $$R=\frac{I_{L}-I_{D}}{P_{i}S}=\frac{I_{P}}{P_{i}S}$$

where *I_L_* is the current under UV light illumination, *I_D_* is the dark current, *I_P_* is the photocurrent, *P_i_* is the power density of the incident light, and *S* is the effective photoresponse area (0.2 cm^2^ for this experiment).

On the other hand, detectivity can be determined with Equation (8):

(8) $$D^{*}=\frac{\sqrt{A}\cdot R}{\sqrt{2qI_{D}}}$$

where *A* is the active area of the photodetector, *R* is the responsivity, *q* is the charge constant, and *I_D_* is the dark current of the device.

Equations (7) and (8) show that *R* and D* can increase by increasing *I_L_* or decreasing *I_D_*. *I_D_* can decrease due to the current-rectifying characteristic of the p-n heterojunction [30]. On the other hand, *I_L_* can increase through flowing multiple charges during the lifetime of photoelectrons due to the higher in-plane carrier mobilities of h-BN than those in the vertical direction [31,32]. The two points discussed above explain the high photoresponse and detectivity of the UV-PDs of VO-hBN/GaN.

**Figure 9:**
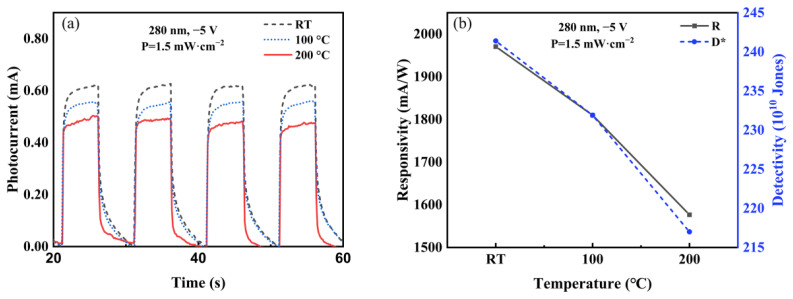
(**a**) Photocurrent as a function of time measured at different temperatures under −5 V bias voltages under illumination of 1.5 mW/cm^2^ for 280 nm. (**b**) Responsivity and detectivity as a function of temperature under −5 V bias voltages under illumination of 1.5 mW/cm^2^ for 280 nm.

In order to research the stability of the VO-hBN/GaN heterojunction under harsh conditions such as high temperature, the photoelectric property of the VO-hBN/GaN heterojunction was investigated under different temperatures, as shown in Figure 9 (1.5 mW/cm^2^, 280 nm, −5V). The results show that the photocurrent, responsivity, and detectivity decreased 20.0%, 20.0%, and 10.3% at 200 °C, compared with those at room temperature (RT). This acceptable stability may benefit from the high in-plane heat dissipation performance of hBN.

## 4. Conclusions

In summary, vertically oriented BN films were successfully synthesized directly in GaN by using boron and ammonia gas as the sources of boron and nitrogen, respectively. The whole preparation process was achieved using economical furnace equipment, which overcame the disadvantage of complex and expensive equipment in conventional preparation methods. Additionally, at the same time, the synthesis process, without transfer and a catalyzer, could avoid contamination. The intrinsic mechanism model of the vertically self-ordered orientation of h-BN is proposed. The optothermal Raman technique, which has no limitation of specified dimensions with well-defined thickness and is suitable for thin film materials, was used to investigate the thermal conductivity of as-grown GaN and h-BN/GaN samples for the first time. Twice the enhancement of thermal conductivity after the synthesis of vertically oriented h-BN on GaN was found. A stable and high-performance UV-PDs based on the VO-hBN/GaN heterojunction, with photoresponsivity up to 1970.7 mA/W and detectivity up to 2.6 × 10^13^ Jones, was prepared. This work provides a new approach to synthesize h-BN directly on Ⅲ-Ⅴ nitrides, and shows its great application potential by constructing a novel heterojunction structure for photodetection.

## Figures and Tables

**Table 1:** The average temperature coefficients and the power coefficients of Raman peak position for the As-GaN and B/GaN1100 samples.

Sample	*dω/dT* (cm^−1^/°C) E_2_(high) (GaN)	*dω/dP* (cm^−1^/mW) E_2_(high) (GaN)	*K* (W·m^−1^K^−1^)
B/GaN1100	0.016	0.091	425
As-GaN	0.013	0.180	207

**Table 2:** The thermal diffusivity measured using the laser flash technique for the As-GaN and B/GaN1100 samples.

Sample	Diffusivity at 25 °C (mm^2^/s)	Diffusivity at 200 °C (mm^2^/s)	Diffusivity at 400 °C (mm^2^/s)
B/GaN1100	7.199	10.373	15.123
As-GaN	5.367	9.120	12.837

## Data Availability

The data presented in this study are available on request from the corresponding author.
